# WXJ-202, a novel Ribociclib derivative, exerts antitumor effects against breast cancer through CDK4/6

**DOI:** 10.3389/fphar.2022.1072194

**Published:** 2023-01-19

**Authors:** Jing Ji, Wenwen Liu, Yuxin Xu, Zhou Xu, Mingxiao Lv, Jing Feng, Jinyu Lv, Xingbei He, Zhen Zhang, Mengru Xie, Aixin Jing, Xiujun Wang, Jinming Ma, Bin Liu

**Affiliations:** Jiangsu Key Laboratory of Marine Pharmaceutical Compound Screening, College of Pharmacy, Jiangsu Ocean University, Lianyungang, China

**Keywords:** breast cancer, molecular docking, *CDK4/6*, cell cycle, apoptosis

## Abstract

Cyclin-dependent kinases 4 and 6 (*CDK4/6*) are key regulatory proteins in the cell division and proliferative cycle in humans. They are overactive in many malignant tumors, particularly in triple-negative breast cancer (TNBC). Inhibition of *CDK4/6* targets can have anti-tumor effects. Here, we designed and synthesized a novel derivative of Ribociclib that could affect *CDK4/6*, named WXJ-202. This study aimed to investigate the effects of compound WXJ-202 on proliferation, apoptosis, and cell cycle arrest in human breast cancer cell lines and their molecular mechanisms. We assayed cell viability with methyl thiazolyl tetrazolium (MTT) assay. Clone formation, migration, and invasion ability were assayed by clone formation assay, wound healing assay, and transwell invasion assay. The effect of compound WXJ-202 on apoptosis and cell cycle was detected by flow cytometry analysis. Western blotting was performed to detect the expression of proteins related to the *CDK4/6*-*Rb*-*E2F* pathway. The anti-cancer effects were studied *in vivo* transplantation tumor models. WXJ-202 was shown to inhibit cell proliferation, colony formation, migration, and invasion, as well as induce apoptosis and cycle arrest in breast cancer cells. The levels of proteins related to the *CDK4/6*-*Rb*-*E2F* pathway, such as *CDK4*, *CDK6*, and *p-Rb*, were decreased. Finally, studies had shown that compound WXJ-202 exhibited significant anti-tumor activity in transplantation tumor models. In this research, the compound WXJ-202 was shown to have better anti-tumor cell proliferative effects and could be used as a potential candidate against TNBC tumors.

## 1 Introduction

Breast cancer is among the most common malignancies in women (Wang et al., 2021; Sofi et al., 2022). Breast cancer is primarily a malignant lesion caused by abnormal differentiation of the epithelial tissue of the breast (Sun et al., 2018). Patients’ physical and mental health is seriously threatened by breast cancer. According to statistics, about 19.3 million new cancer patients were diagnosed in 2020 worldwide, of which breast cancer in women accounted for 11.7%, surpassing lung cancer (11.4%) for the first time in terms of numbers, making it the most newly diagnosed cancer in the world (Sung et al., 2021). In terms of mortality, there were 10 million deaths from cancer in 2020, of which 685,000 were due to breast cancer (Kufel-Grabowska et al., 2022). Breast cancer has much higher morbidity rate than other cancers, so people should take breast cancer seriously (Ahmad, 2019). Triple-negative breast cancer (TNBC) is a subtype of breast cancer (Dukhanina et al., 2022). The disease is characterized by high invasiveness, poor responsiveness to therapy, and difficulty with treatment (Medina et al., 2020; Luna-Dulcey et al., 2020). TNBC is still primarily treated with chemotherapeutics postoperatively due to a lack of effective therapeutic targets (Walsh et al., 2022). Some patients, however, have difficulty tolerating chemotherapy, and once distant metastasis has occurred, the duration of survival and quality of life is severely reduced (Wang et al., 2022). Therefore, there is an urgent need to explore novel drugs for the treatment of TNBC, which has also been a difficult and hot topic of research in recent years.

The cyclin-dependent kinases (*CDKs*) of the serine/threonine protein kinase family include transcriptional regulators (mainly *CDK7*, *CDK8*, *CDK9*, *CDK10*, *CDK11*, etc.) and cell cycle regulators (primarily *CDK1*, *CDK2*, *CDK4*, *CDK6*, etc.) (Pandey et al., 2022). Dysregulation of the activity of *CDKs* can directly or indirectly lead to uncontrolled cell proliferation, such as genomic instability (increased DNA mutations, chromosomal deletions, etc.), as well as chromosomal instability (changes in chromosome number) to participate in tumor development (Ettl et al., 2022). *CDKs* have a strong influence on the regulation of the tumor cell cycle, and disruption of the tumor cell cycle can impact cell division and growth (Duster et al., 2022). In the G1-S-G2-M cell cycle, *CDK4/6* is a crucial functional protein that binds to *Cyclin D1* to form the *cyclin D1*-*CDK4/6* complex, which releases the transcription factor *E2F* by phosphorylating the retinoblastoma protein (*RB*) (Arnold and Papanikolaou, 2005; Schachter et al., 2013). This promotes transcription of the associated genes, allows the cell to progress from G1 to the S phase, and enables the cell cycle to proceed (Spring et al., 2019). Overexpression of *CDK4/6* leads to abnormalities in the *CDK4/6*-*Rb*-*E2F* pathway, which is an essential cause of the development of breast cancer (Lin et al., 2019). Inhibitors of *CDK4/6* can arrest tumor cell development, inhibit the over-proliferation of tumor cells, and prevent abnormal tumor cell replication (Braal et al., 2021). It selectively inhibits *CDK4/6* and arrests the associated tumor cells from moving from G1 to the S phase (Ingham and Schwartz, 2017). Thus, targeted drug design for the treatment of breast cancer with *CDK4/6* as a target has important potential. Three selective *CDK4/6* inhibitors have been approved by the FDA, including Palbociclib, Ribociclib, and Abemaciclib (Pandey et al., 2019). Previous research has found IC50 values for these drugs in the low nanomolar range for *CDK4/6* (Roskoski, 2019). Currently, three *CDK4/6* inhibitors have been approved in combination with hormonal therapies for the treatment of breast cancer patients, but patients are prone to develop drug resistance with long-term use (Papadimitriou et al., 2022).

In this study, we designed and synthesized a novel Ribociclib derivative, 2-((3-chloro-4-(pyridin-2-ylmethoxy) phenyl) amino)-7-cyclopentyl-N, N-dimethyl-7H-pyrrolo [2,3-d] pyrimidine-6-carboxamide (WXJ-202). We speculated that WXJ-202 and Ribociclib may have similar inhibitory effects against TNBC. Based on the molecular docking results, compound WXJ-202 was found to have a high binding capacity for *CDK4* and *CDK6*. This experiment explored the possible anti-tumor effect of compound WXJ-202 on TNBC as well as its molecular mechanism, which provided a potential drug target for the treatment of TNBC.

## 2 Materials and methods

### 2.1 Chemistry

The following instruments were used: electronic balance (Hua Zhi Electronic Technology Co., Ltd.), 45–75 μM silica gel powder for column chromatography, silica gel GF254 for thin layer chromatography (TLC), UV lamp (model UV-IIB) for color development, Q Exactive HF LC-MS (Thermo Fisher Scientific) for mass spectrometry, Avance III 500 MHz liquid nuclear magnetic resonance spectrometer (TMS as internal standard, Bruker, Germany). All other reagents were analytically pure and used directly without further processing.

### 2.2 Molecular modeling methods

All molecular docking research were conducted on a Viglen Genie Intel (R) Core (TM) i5-7300HQ CPU @ 2.50 GHz running Windows 10 1909. AutoDockTools-1.5.6 and Pymol-1.7.0.0.win 32-py2.7 as software for molecular modeling. The X-ray co-crystallized structure of *CDK4* and *CDK6* were downloaded from the PDB data bank (http://www.rcsb.org/) and used for docking studies. The proteins were pretreated with Pymol to remove HOH and metal ions. Ligand structures were constructed using Chem3D. The docking results were visually inspected to determine the binding ability of the analyzed compounds at the active site.

### 2.3 Cell culture

The human cancer cell lines MDA-MB-231, MCF-7, A498, and Hela cells were purchased from the National Biomedical Laboratory (Beijing) and cultured in DMEM (KGM12800-500) or MEM medium (KGM41500-500) containing 10% fetal bovine serum (FBS) and 1% penicillin-streptomycin, and the cells were incubated at 37°C and 5% CO_2_ in a constant temperature incubator (Thermo Fisher Scientific, BB150). 0.25% trypsin was added when the confluence of cells was 70%–80% for digestion, passagework, and culture. For the next study, cells in the logarithmic growth phase and favorable growth state were selected.

### 2.4 Cell proliferation assay

Inoculated cells into 96-well culture plate with 1 × 10^4^ cells per well. After the cells had adhered, the medium in the 96-well plate was removed, and the cell culture medium with various concentrations of the compound WXJ-202 and the positive drug was added to the various groups, respectively. The cells were then treated for 24 h. 10 μL of methyl thiazolyl tetrazolium (MTT) solution was added to each well and continued to be incubated for 4 h. After the supernatant was removed, 100 μL of dimethyl sulfoxide (DMSO) was added to each well for color development. Each group’s absorbance (OD) was measured at 490 nm using a multifunctional microplate detector (BioTek, SYNERGY Neo2, United States).

The following formula was used to determine the inhibition rate based on the OD values obtained from a multipurpose microplate detector (BioTek, SYNERGY Neo2, United States). If the inhibition rate was ≤0%, it was recorded as 0%.

$$Inhibition\;Rate\;\left(\%\right)=\left(1-\frac{Experimental\;Group\;OD}{Control\;Group\;OD}\right)\times 100\%$$

### 2.5 Cell morphology

The effect of WXJ-202 on MDA-MB-231 cells was determined by morphological changes. MDA-MB-231 cells (1 × 10^5^ cells/well) were inoculated into 12-well plates and kept overnight at 37°C and 5% CO_2_. After that, the cells were exposed to different concentrations of WXJ-202, and positive drugs, respectively. Images were taken with an inverted microscope (Gangnam XD-202).

### 2.6 Cell adhesion assay

The MDA-MB-231 and MCF-7 cells were plated in 12-well culture plates and cultivated for 24 h at 37°C with 5% CO_2_, respectively. After aspirating and discarding the supernatant, the medium containing different concentrations of the compound WXJ-202 and the positive drug was added, respectively, and the mixture was incubated for 24 h. After collecting cells by centrifugation (1000 rpm, 5 min), cells were counted and inoculated at 10,000 cells per well in a 96-well plate that had been pre-coated with matrix gel (Corning Biocoat, 354248). The supernatant was removed after 1 h and 10 μL of MTT solution was added to continue the incubation for another 4 h. The supernatant was discarded and 100 μL of dimethyl sulfoxide (DMSO) was added to each well for color development. Each group’s absorbance (OD) values were then measured at 490 nm using a multifunctional microplate detector (BioTek, SYNERGY Neo2, United States) to calculate the adhesion rate.

### 2.7 Wound healing scratch assay

MDA-MB-231 and MCF-7 cells were inoculated into 12-well cell culture plates at a density of 1.5 × 10^5^ cells/well, respectively. Overnight incubation allowed them to grow as a single cell layer. Scratch over the cell layer in the central portion of each well with a sterile pipette tip to create a scratch. Following that, cells were cleaned of cellular debris using sterile PBS. Cells were incubated with different concentrations of the compound WXJ-202 or positive drug in a complete medium. Images were taken at 0, 24 and 48 h using an inverted optical microscope (Gangnam XD-202). Observed the healing of scratches with time. The percentage of wound closure at 24 and 48 h was estimated from the closed area of migratory cells by using ImageJ software. The formula for calculating the scratch healing rate was as follows.

$$Scratch\;Healing\;Rate\;\left(\%\right)=\frac{0h\;Scratch\;Area-Time\;Point\;Scratch\;Area}{0h\;Scratch\;Area\;}\times 100\%$$

### 2.8 Cell invasion assay

The matrix gel (Corning Biocoat, 354248) was pre-thawed overnight at 4°C and diluted to 200 μg/mL using DMEM serum-free medium according to the instructions. 100 μL was wrapped on the bottom of the upper chamber surface of the Transwell and air-dried at 37°C and 5% CO_2_ in an incubator for 4 h. Different concentrations of compound WXJ-202 and positive drug were added to MDA-MB-231 and MCF-7 cells, respectively, and the negative control concentration was 0 µg/ml. After 24 h, the cells were collected by centrifugation and prepared into 100 μL single cell suspension using serum-free medium containing 0.2% BSA and added dropwise to the upper chamber. Meanwhile, 650 µL of medium containing 10% fetal bovine serum (FBS) was added to the lower chamber as a chemotactic invasion inducer. After 24-48 hours of incubation, cells on the surface of the upper chamber of the Transwell were wiped off with a cotton swab. Cells that invaded the lower chamber of Transwell were fixed in paraformaldehyde, washed with PBS after 15 min, and stained with Giemsa or Crystal violet for 30 min. The staining solution was washed off with deionized water, dried, and placed under an inverted light microscope (Gangnam XD-202) to photograph randomly selected areas. Invasive cells were counted with ImageJ software.

### 2.9 Colony formation assay

The logarithmic growth phase MDA-MB-231 cells were inoculated into 6-well culture plates and MCF-7 cells were inoculated into 12-well plates, adding 1,000 cells of logarithmic growth stage to each well. The cells were evenly dispersed and incubated at 37°C in a 5% CO_2_ incubator. After the cells were plastered, a culture medium containing different concentrations of compound WXJ-202 or positive control drug was added to each well, while a negative control group was set up. After 12 h of treatment, the medium was replaced with the normal medium, and the culture was continued for another 2 weeks with medium changes every 3 days. After the clones were formed, they were washed with PBS and fixed with 4% paraformaldehyde. The plates were then stained with Giemsa or Crystal violet, dried, and observed for colony formation. Finally, the well plates were inverted, photographed, and counted with ImageJ software.

### 2.10 Annexin V/Propidium iodide (PI) staining

The MDA-MB-231 cells were inoculated into 6-well plates at a density of 5 × 10^5^ cells per well and incubated at 37°C with 5% CO_2_. The following day, different concentrations of compound WXJ-202 and positive drugs were added, respectively. After a 24 h treatment period, cells were collected by trypsinization without EDTA and centrifuged at 1,000 rpm for 10 min. After washing twice with PBS by gentle blowing, incubated with 10 μL Annexin V-FITC solution and 5 μL propidium iodide staining solution for 15 min at room temperature under light-proof conditions. The apoptosis rate of each group of cells was detected on a Guava flow cytometer (Guava easycyte 6HT 2L) and analyzed by Flowjo software.

### 2.11 Cell cycle analysis

The MDA-MB-231 cells were seeded in 6-well plates at 1 × 10^6^ cells per well. After the cells adhered, the serum was withdrawn for 24 h to synchronize the cells. A fresh culture medium containing different concentrations of compound WXJ-202 or positive drugs was replaced for 24 h treatment. The cells were digested with 0.25% trypsin and collected by centrifugation at 800 rpm for 5 min. 500 μL of pre-cooled phosphate buffered solution (PBS) was used to resuspend the cells twice, each time at 800 rpm for 5 min. PBS was discarded and the cells were fixed in 70% pre-cooled anhydrous ethanol at 20°C for 12–24 h. After being centrifuged and resuspended for 5 min, the fixed cells underwent three PBS washes. The supernatant was discarded and RNaseA was added for 30 min at 37°C. Finally, 500 μL of propidium iodide (PI) was added and stained for 30 min at room temperature and protected from light, and the cell cycle was measured using a flow cytometer (Guava easycyte 6HT-2 L) and analyzed using Flowjo software.

### 2.12 Western blot analysis

The MDA-MB-231 cells were inoculated in 6-well plates and treated with different concentrations of compound WXJ-202 or positive drugs after the cells adhered. After culturing in a cell incubator for 24 h, the cells of each group were collected. The cells were lysed with the cell lysis solution. After high-speed centrifugation, the supernatant was extracted and the protein was quantified by the BCA method. An appropriate amount of protein was taken, electrophoresed in 1 × SDS-PAGE buffer, and transferred to the methanol-pretreated PVDF membrane by the “wet transfer method.” The membrane was closed with 5% skimmed milk for 45 min, followed by adding primary antibody dilution (1:1,000 volumetric dilution): *CDK4* (beyotime, AF6465), *CDK6* (beyotime, AF0114), *Cyclin D1* (ABclonal, A11022), *E2F1* (beyotime, AF6756), *Rb* (beyotime, AF1564), *p-Rb* (beyotime, AF1135), *Caspase-3* (beyotime, AF1213), *Pro-caspase-3* (beyotime, AF1261), *β-actin* (beyotime, AA128). Incubated overnight at 4°C on a shaker. The next day, the membrane was washed 4 times with 1 × PBST, 5 min/time, and the corresponding secondary antibody diluent (volume dilution ratio 1:10,000) was added, and incubated at room temperature for 45 min. Washed the membrane 4 times with 1 × PBST, 5 min/time. Dropping luminescent liquid to develop on the imaging system. Data were processed using ImageJ software.

### 2.13 Chorio-allantoic membrane model of chicken embryo

The breeding eggs were purchased from the Wufengshan Chicken Farm in Tongling, Anhui, China. All animal experiments followed the standards of the Animal Ethics Committee. Breeding eggs were incubated in a sterile environment at 37°C for 7 days, and then the air chamber was transferred to 1 cm from the umbilical blood vessel. The windows were sealed with sterile plastic wrap for later use. The cells were resuspended using a mixed suspension (1:1) of Matrigel and DMEM and adjusted them to 1.2-1.5 × 10^6^ cells/mL. resuspended cells were implanted into the vessels. After 4 days of implantation, a negative control group (DMSO), a positive control group and the experimental group were set up with 6 chicken embryos per group (*n* = 6). Different concentrations of WXJ-202 and positive drugs were added to the chicken embryo allantoic membrane, respectively. After 3 days of treatment with the drug, the tumors were removed, photographed, weighed, and recorded.

### 2.14 Analytical statistics

GraphPad Prism 9 software was used for the statistical analysis, and the data were expressed as the mean ± S.D of three independent experiments. The t-test was used to compare the data between the two groups. Statistics were considered significant for *p* values under 0.05.

## 3 Results

### 3.1 Synthetic route of compound WXJ-202

2-((3-chloro-4-(pyridin-2-ylmethoxy) phenyl) amino)-7-cyclopentyl-N, N-dimethyl-7H-pyrrolo [2,3-d] pyrimidine-6-carboxamide (WXJ-202).

**FIGURE 1:**
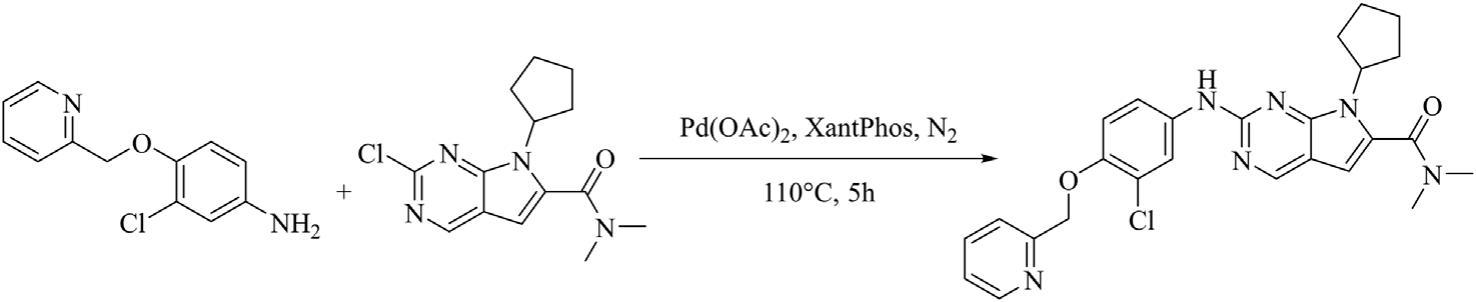
Synthetic route of compound WXJ-202.

Under nitrogen protection, a 250 mL round bottom flask was charged with 2-chloro-7-cyclopentyl-N,N-dimethyl-7H-pyrrolo [2,3-d]pyrimidine-6-carboxamide(0.293 g, 1 mmol),3-chloro-4-(2-pyridylmethoxy) aniline (0.235 g, 1 mmol), Pd(OAc)_2_ (0.0565 g, 0.25 mmol), Cs_2_CO_3_ (0.978 g, 3.0 mmol), XantPhos (0.289 g, 0.5 mmol) and 1,4-dioxane (25 mL), under the condition of 110°C, after stirring reaction for 5 h, monitoring should be until the raw material point disappears (developing solvent: dichloromethane: methanol = 40:1) under reduced pressure to remove the solvent to give a dark brown oil. Through rapid column chromatography separation (200–300 mesh silica gel, dichloromethane: methanol = 80: 1), the target product (0.278 g) was obtained as a pale-yellow solid with a yield of 56.6% (Figure 1). Hydrogen spectrum of compound WXJ-202 (Supplementary Figure S1): ^1^H NMR (500 MHz, Chloroform-*d*) δ 8.62 (s, 1H), 8.59 (d, *J* = 4.8 Hz, 1H), 8.06 (d, *J* = 2.6 Hz, 1H), 7.75 (td, *J* = 7.7, 1.6 Hz, 1H), 7.67 (d, *J* = 7.8 Hz, 1H), 7.41 (d, *J* = 21.6 Hz, 1H), 7.26–7.20 (m, 2H), 6.94 (d, *J* = 8.9 Hz, 1H), 6.42 (s, 1H), 5.29 (d, *J* = 11.8 Hz, 2H), 4.73 (s, 1H), 3.15 (s, 6H), 2.65–2.46 (m, 2H), 2.14–2.00 (m, 4H), 1.67 (q, *J* = 6.1 Hz, 2H); Carbon spectrum of compound WXJ-202 (Supplementary Figure S2): ^13^C NMR (126 MHz, Chloroform-*d*) δ 164.10, 157.11, 155.41, 152.00, 151.71, 149.04, 148.87, 136.95, 134.70, 131.96, 123.13, 122.66, 121.25, 121.19, 118.24, 114.39, 112.43, 100.91, 71.84, 58.13, 30.15, 24.41; HR-MS *m/z*: 491.1965[M + H]^+^.

### 3.2 Molecular docking of compound WXJ-202

**FIGURE 2:**
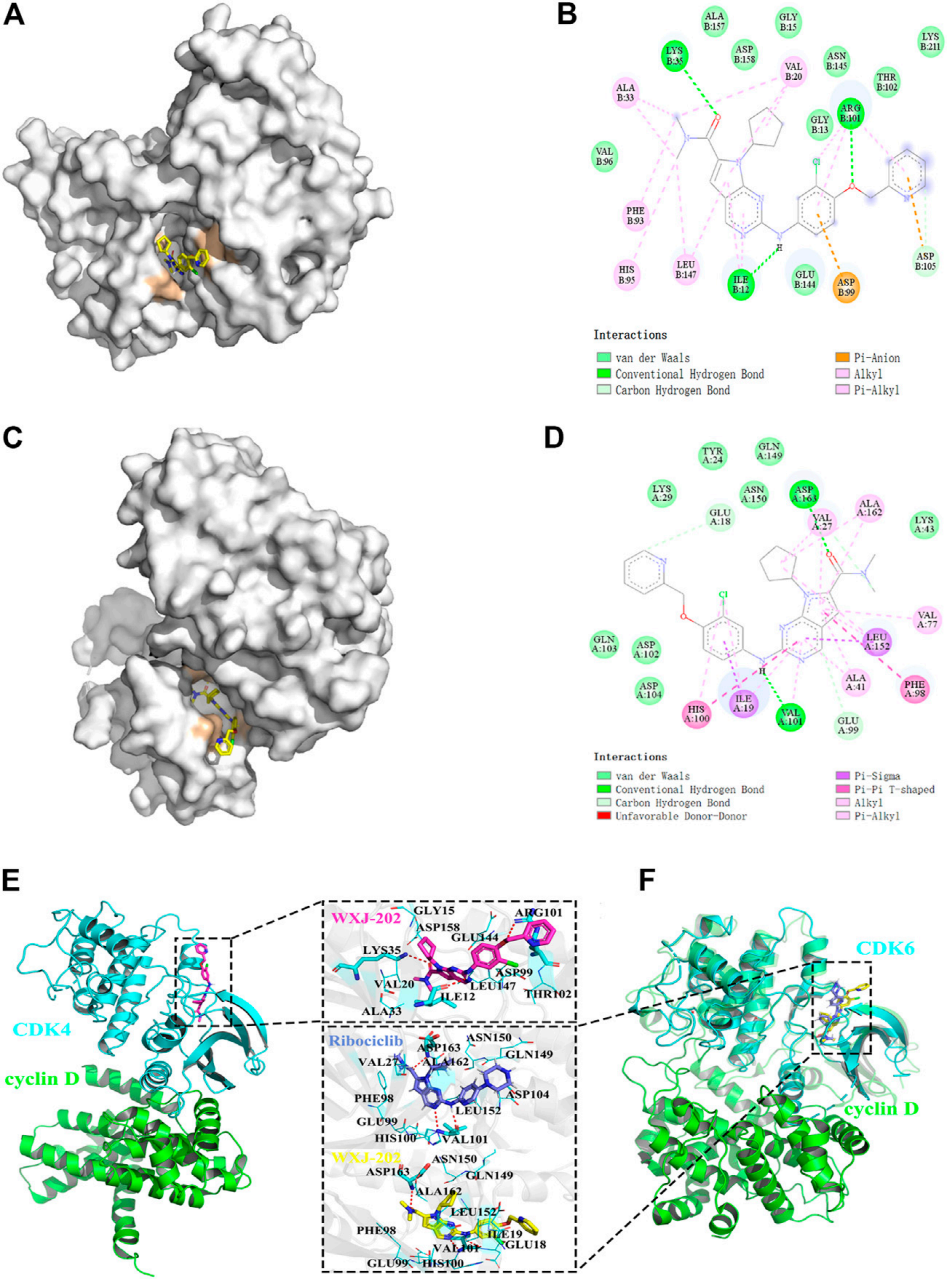
Molecular docking between compound WXJ-202 with *CDK4* and *CDK6.* **(A)** Site of docking binding of compound WXJ-202 in *CDK4* protein kinase. **(B)** 2D interaction map of compound 202 with *CDK4* protein kinase. **(C)** Position of the compound 202 binding in *CDK6* protein kinase. **(D)** 2D interaction map of compound 202 with *CDK6* protein kinase. **(E)** Interactions between the CDK4 active binding site and compound WXJ-202. **(F)** Interactions between the CDK6 active binding site and compound WXJ-202. Complex model of CDK4 (blue) and Cyclin D (green) was taken from the PDB database (ID: 2W9Z). The complex model of CDK6-Cyclin D (green) was taken from the PDB database (ID: 1XO2). As a reference, the crystal structure of CDK6-Ribociclib (blue, ID:5L2T) was also aligned to the protein-protein complex. Among, the residues forming hydrogen bonds with the inhibitor were shown as blue sticks, and the remaining residues were shown as blue lines, the red dashed lines indicate hydrogen bonds.

To investigate the binding of *CDK4* and *CDK6* to the compound WXJ-202, the program AutoDockTools-1.5.6 was used to perform a molecular docking study. The position of docking binding of compound WXJ-202 in *CDK4* protein kinase (Figure 2A) indicated that compound WXJ-202 bound tightly to *CDK4* and generated three hydrogen-bonding forces. The 2D interaction diagram of compound WXJ-202 with *CDK4* protein kinase (Figure 2B) showed that these three hydrogen bonding forces were -CO- with LYS 35, -NH with ILE 12, -O- with ARG101. The location of compound WXJ-202 binding in *CDK6* protein kinase (Figure 2C) revealed that the pyridine end of compound WXJ-202 extended into a groove on the outside of the pocket. The 2D interaction diagram of compound WXJ-202 with *CDK6* protein kinase (Figure 2D) revealed that compound WXJ-202 had a significant interaction force with *CDK6* protein kinase. This included strongly molecularly interacting hydrogen bonds, where -NH interacted with VAL 101 *via* hydrogen bonding and -CO- interacts with ASP163. The molecular docking results showed that both *CDK4* and *CDK6* were strongly bound to the compound WXJ-202.

In addition, we investigated the complexes formed by *CDK4* and *CDK6* with *Cyclin D*. Meanwhile, the binding conformations of WXJ-202 and Ribociclib in the binding pocket of *CDK6* were superimposed for a more visual comparison. The results (Figures 2E, F) indicated that both Ribociclib and 202 maintained a similar conformation in the same binding pocket.

### 3.3 Anti-proliferative activity of compound WXJ-202

To explore the *in vitro* antitumor cell activity of compound WXJ-202, we performed MTT assays. The toxic effects of different concentrations of compound WXJ-202 on MDA-MB-231, A498, MCF-7, and Hela cell lines were examined using Abemaciclib, Ribociclib, and Imatinib as positive controls, respectively. The results are shown in Table 1. The results showed that after 24 h of treatment, compound WXJ-202 showed a progressive increase in inhibition of these 4 cell types compared to the control. The IC50 values calculated from the analysis are shown in Table 1. Compound WXJ-202 showed IC50 values of (9.31 ± 1.82 μM) on MDA-MB-231 cells, (7.16 ± 0.71 μM) on A498 cells, (5.76 ± 0.46 μM) on MCF-7 cells, and (3.64 ± 0.18 μM) on Hela cells, all superior to Ribociclib and Imatinib. Meanwhile, the IC50 of compound WXJ-202 on MDA-MB-231 cells (9.31 ± 1.82 μM) was lower than that of Imatinib (37.12 ± 3.18 μM), Ribociclib (34.96 ± 3.87 μM) and Abemaciclib (15.04 ± 4.53 μM).

**TABLE 1:** The cytotoxic effects of compound WXJ-202 and positive drugs (abemaciclib, ribociclib, and imatinib) on MDA-MB-231, MCF-7, A498, and Hela cell lines.

Compound	IC50(μM)			
	MDA-MB-231	MCF-7	A498	Hela
WXJ-202	9.31±1.82	5.76±0.46	7.16±0.71	3.64±0.18
Abemaciclib	15.04±4.53	6.36±1.45	15.93±1.82	3.65±1.12
Ribociclib	34.96±3.87	15.71±3.28	24.73±3.73	16.68±2.64
Imatinib	37.12±3.18	19.54±3.98	31.85±4.14	25.11±3.71

Data were expressed as the mean ± SE from the dose-response curves of at least three independent experiments with three determinations in each.

**FIGURE 3:**
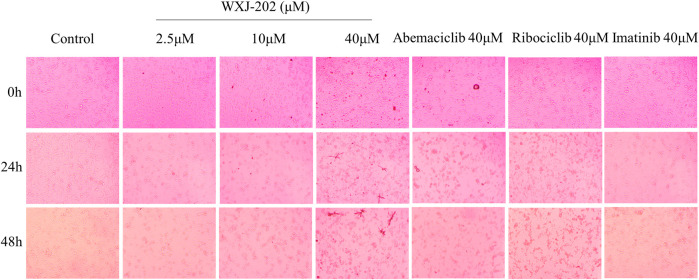
Effects of different concentrations of compound WXJ-202 on the morphology of MDA-MB-231 cells.

Additionally, as demonstrated in Figure 3. By comparing the results of different concentrations of treatment at 0, 24 and 48 h, it was found that the morphology of MDA-MB-231 cells gradually rounded and was accompanied by loss of density at low, medium, and high concentrations (2.5, 10, and 40 μM) as the duration of action increased. The results indicated that the compound WXJ-202 disrupted cell morphology after acting on MDA-MB-231 cells.

### 3.4 Compound WXJ-202 inhibited the adhesion, migration, and invasion of MDA-MB-231 cells and MCF-7 cells

**FIGURE 4:**
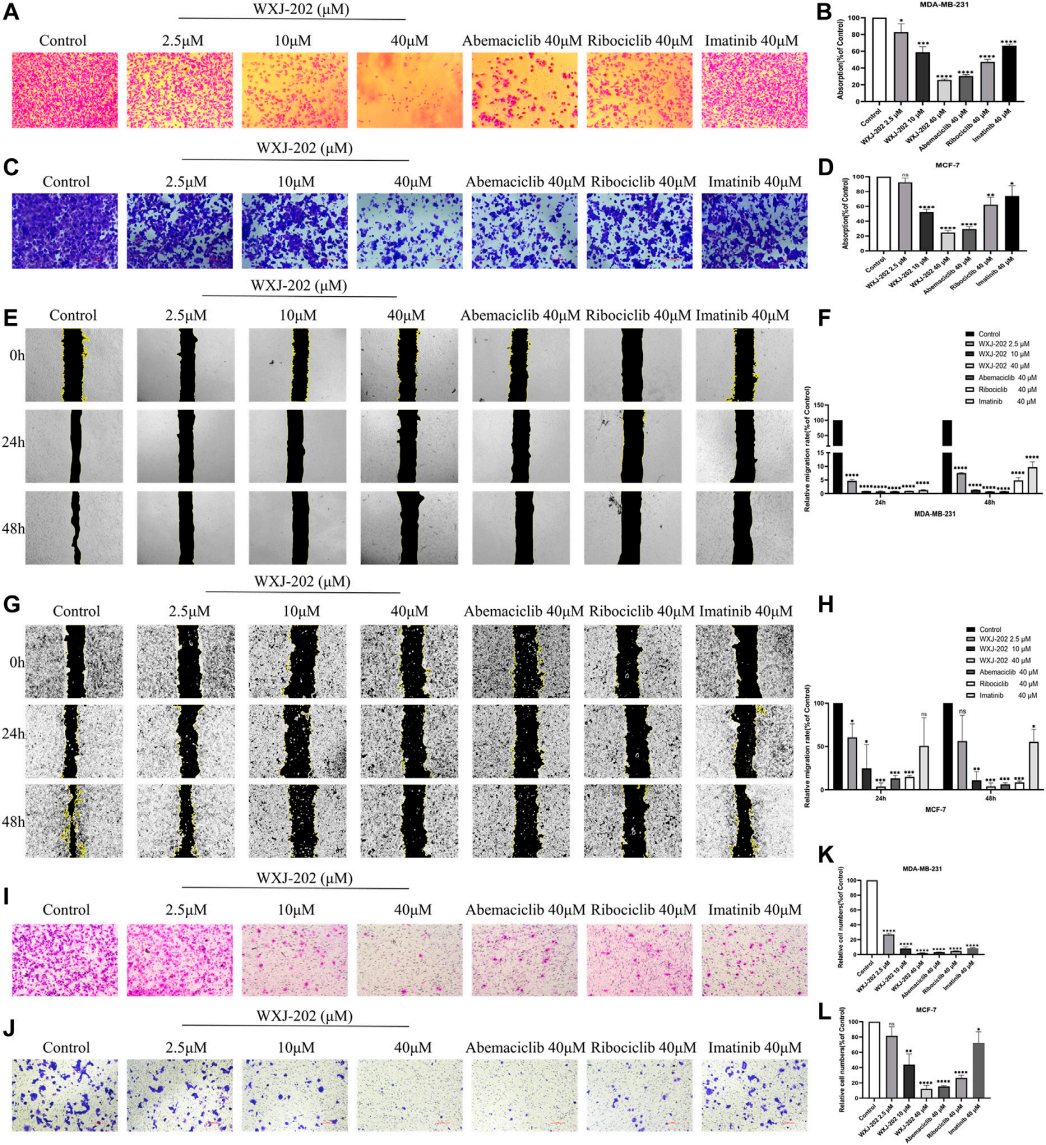
Effects of compound WXJ-202 on the adherence, migration, and invasion of MDA-MB-231 cells and MCF-7 cells. **(A–D)** Effects of compound WXJ-202, abemaciclib, ribociclib and imatinib on the adherence ability of MDA-MB-231 cells and MCF-7 cells. **(E–H)** The effect of different concentrations of compound WXJ-202, abemaciclib, ribociclib and imatinib on the migration ability of MDA-MB-231 cells and MCF-7 cells. **(I–L)** Effects of compound WXJ-202, abemaciclib, ribociclib and imatinib on the invasive ability of MDA-MB-231 cells and MCF-7 cells. *p < 0.05 vs. control, **p < 0.01 vs. control, ***p < 0.001 vs. control, ****p < 0.0001 vs. control.

Preliminary results from cytotoxicity assays indicated that compound WXJ-202 had cytotoxic potential in MDA-MB-231 cells and MCF-7 cells. Therefore, the effect of the compound WXJ-202 on the capacity of MDA-MB-231 cells and MCF-7 cells for adhesion, migration, and invasion was further investigated in this work. By examining the ability of compound WXJ-202-treated cells to adhere, we found that compound WXJ-202 inhibited the ability of MDA-MB-231 and MCF-7 cells to adhere most significantly compared to Ribociclib and Imatinib at the same concentration (Figures 4A–D). Compared with the control group, low, medium, and high concentrations of compound WXJ-202 all showed significant inhibition of the migratory ability of MDA-MB-231 and MCF-7 cells (Figures 4E–H). And this inhibition was stronger than the effect of Ribociclib and Imatinib at the same concentration. Cell invasion assays could assess whether compounds and positive agents could reduce the ability of tumor cells to degrade ECM proteins and invade. In this study, after treatment with 2.5, 10, 40 μM of compound WXJ-202 compared to the control, the number of MDA-MB-231 and MCF-7 cells invading the bottom surface of the transwell membrane was significantly reduced, cell density was significantly lower, cell invasion was significantly reduced, and dose-dependent (Figures 4I–L). The inhibition rates of invasive cells in the compound WXJ-202 low, medium and high concentration groups were (72.66 ± 2.34%, 18.33 ± 11.80%), (92.84 ± 3.46%, 56.11 ± 13.99%) and (97.88 ± 0.62%, 87.98 ± 4.73%), respectively, and the inhibition effects were all more significant than the same concentration of Ribociclib (95.50 ± 0.64%, 73.35 ± 3.45%) and Imatinib (91.30 ± 0.87%, 27.63 ± 14.47%). The above findings indicated that compound WXJ-202 significantly inhibited the adhesion, migration, and invasion of MDA-MB-231 cells and MCF-7 cells.

### 3.5 Compound WXJ-202 inhibited the MDA-MB-231 and MCF-7 colony formation

**FIGURE 5:**
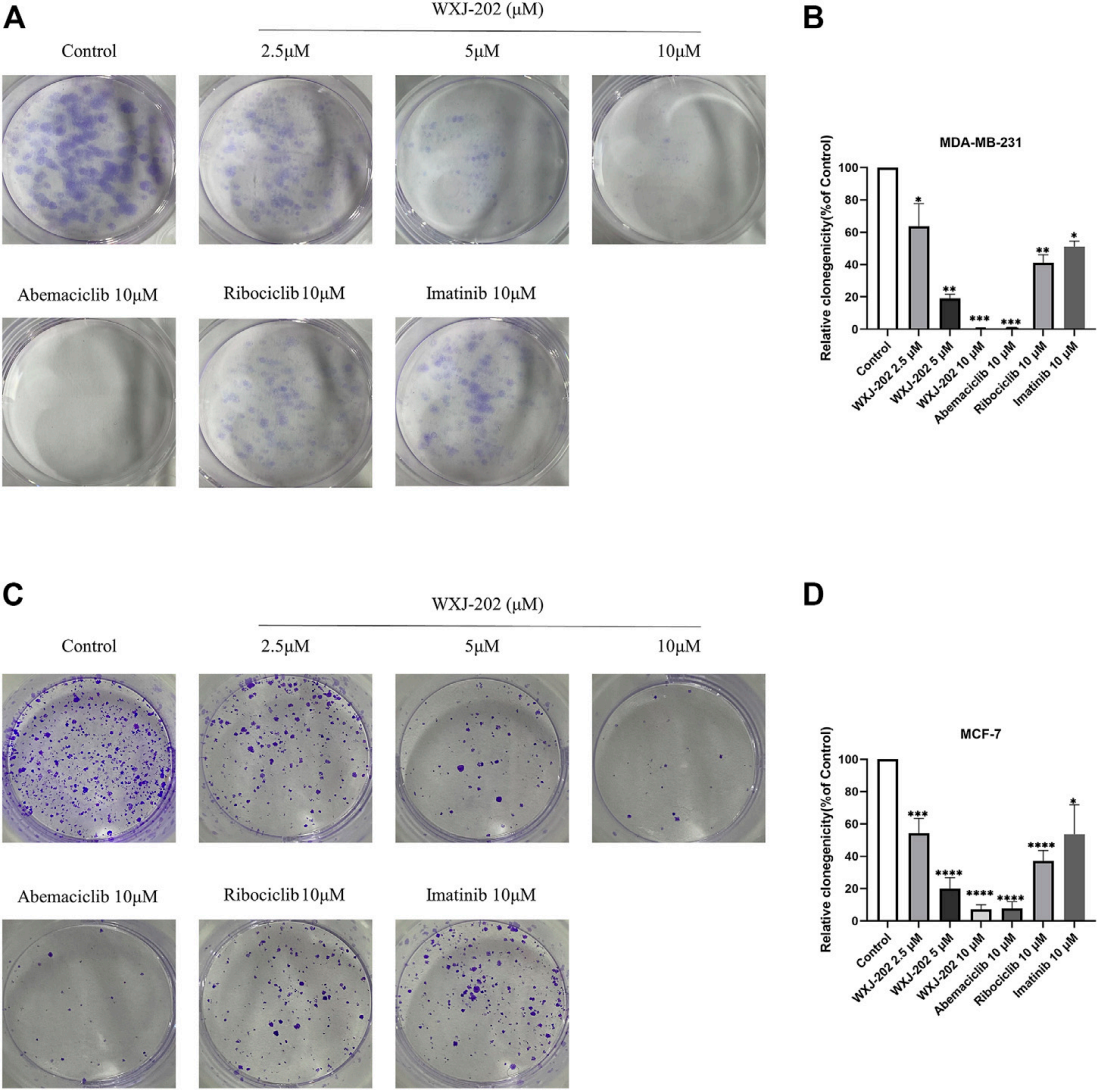
Effects of compound WXJ-202 on colony formation in MDA-MB-231 cells and MCF-7 cells. **(A,B)** Effects of compound WXJ-202, abemaciclib, ribociclib and imatinib on the proliferative capacity of MDA-MB-231 cells. **(C,D)** The effect of compound WXJ-202, abemaciclib, ribociclib and imatinib on the proliferative capacity of MCF-7 cells. *p < 0.05 vs. control, **p < 0.01 vs. control, ***p < 0.001 vs. control, ****p < 0.0001 vs. control.

Next, we incubated different concentrations of compound WXJ-202 and positive drugs with MDA-MB-231 and MCF-7 cells to form clones, respectively. The experimental results (Figures 5A–D) showed that the proliferative capacity of MDA-MB-231 and MCF-7 cells decreased with increasing concentration of compound WXJ-202. When normalized to the control group, the proliferative capacities of the compound WXJ-202 at low, medium, and high doses were (63.89 ± 13.85%, 54.30 ± 9.16%), (19.04 ± 2.60%, 19.92 ± 6.82%), and (0.00 ± 0.00%, 7.08 ± 2.99%), respectively. Additionally, the number of cell clones was significantly reduced in the compound WXJ-202 5 μM and 10 μM treatment groups compared with the 10 μM positive drugs Imatinib (51.16 ± 3.36%, 53.56 ± 18.33%) and Ribociclib (41.17 ± 4.93%, 37.19 ± 6.42%) treatment groups. Thus, our experimental results showed that compound WXJ-202 significantly reduced the proliferative capacity of MDA-MB-231 and MCF-7 cells.

### 3.6 Compound WXJ-202 induced apoptosis in MDA-MB-231 cells

**FIGURE 6:**
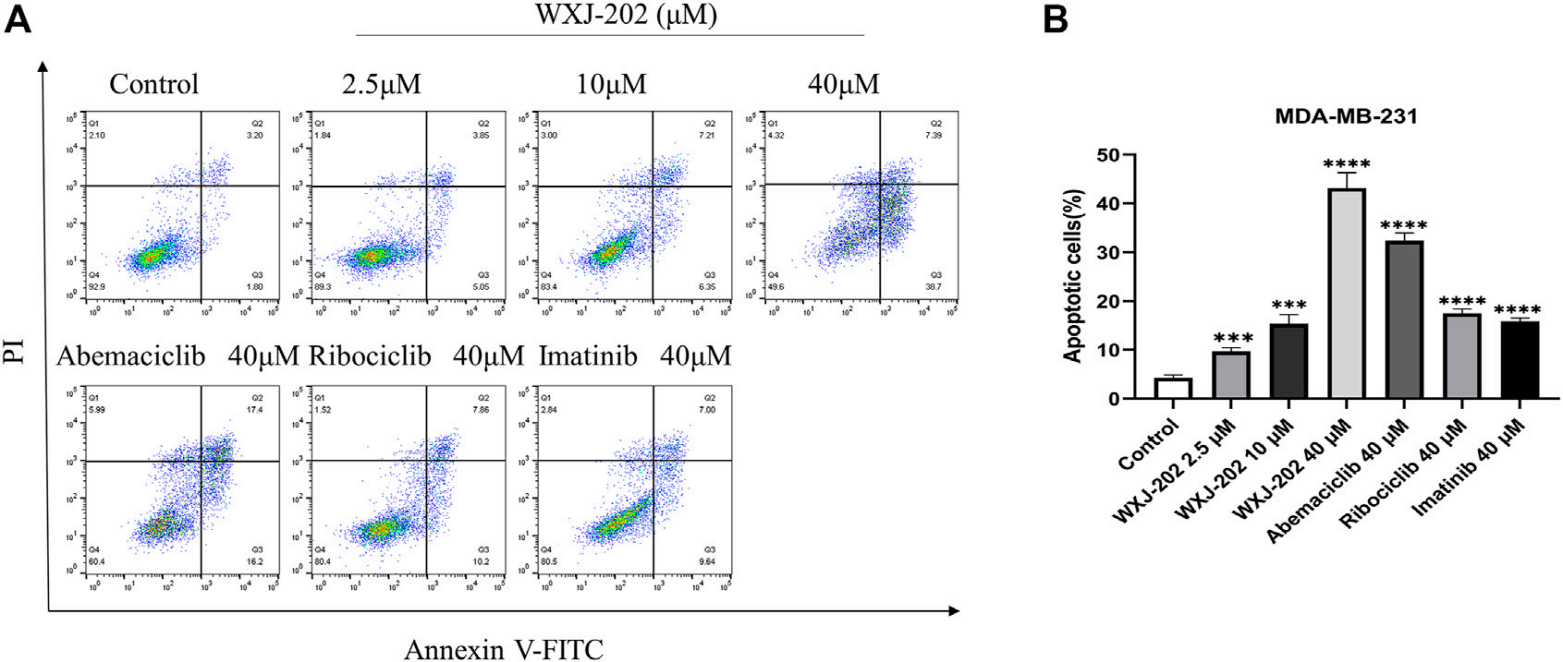
Effects of compound WXJ-202 on the apoptosis of MDA-MB-231 **(A)** Apoptosis analysis with flow cytometry. **(B)** The quantified histogram displayed the apoptosis rate. ***p < 0.001 vs. control, ****p < 0.0001 vs. control.

To determine the impact of compound WXJ-202 on apoptosis in breast cancer cells MDA-MB-231, the AnnexinV-FITC Apoptosis Detection Kit was used to detect MDA-MB-231 cells treated with compound WXJ-202. Our experimental results (Figures 6A, B) showed that the compound WXJ-202 significantly induced apoptosis, and the apoptosis was concentration-dependent. When the compound WXJ-202 concentration was varied from 2.5 μM to 40 μM, the apoptotic rate increased from 8.9% to 46.09%. Next, we used Western blot assay to verify the mechanism of apoptosis induction by compound WXJ-202. The results showed elevated expression levels of apoptosis-associated protein *Caspase-3* and decreased expression levels of *Pro-caspase-3* (Figures 8A, C, D). The above results indicated that compound WXJ-202 could induce apoptosis in MDA-MB-231 cells.

### 3.7 Compound WXJ-202 induced cell cycle arrest in the G0/G1 phase of MDA-MB-231

**FIGURE 7:**
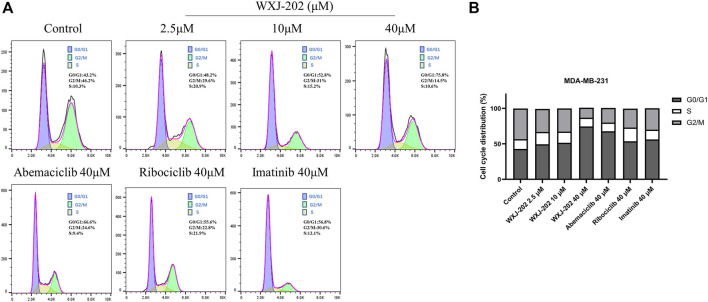
Effects of compound WXJ-202 on the cell cycle of MDA-MB-231 **(A)** Cell cycle analysis with Flow Cytometry. **(B)** The quantified histogram displayed the cell cycle distribution. Data represent the mean ± S.D of at least three independent experiments.

To further explore the mechanism by which the compound WXJ-202 exerts its anti-tumor effects, we analyzed the cell cycle changes after its action on MDA-MB-231 cells by flow cytometry. Compound WXJ-202 treated MDA-MB-231 cells at 2.5, 10 and 40 μM concentrations for 24 h. The ratios of the G0/G1 phase in the compound WXJ-202 group were (48.2 ± 4.8%), (51.9 ± 1.9%) and (75.8 ± 4.7%), respectively. In comparison to untreated cells [negative control group (42.9 ± 0.5%)], G1-phase cells in the compound WXJ-202 group grew by almost 33% at the 40 μM dose. The G0/G1 phase ratios in the positive control Ribociclib group (53.3 ± 2.3%) and the Imatinib group (56 ± 0.8%) were both lower than that of the compound WXJ-202 group at the same concentration (Figures 7A, B). The above experimental results indicated that compound WXJ-202 could significantly induce G0/G1 phase arrest in tumor cells and exert an anti-tumor activity.

### 3.8 Compound WXJ-202 regulated the expression of related cyclin proteins in MDA-MB-231 cells

**FIGURE 8:**
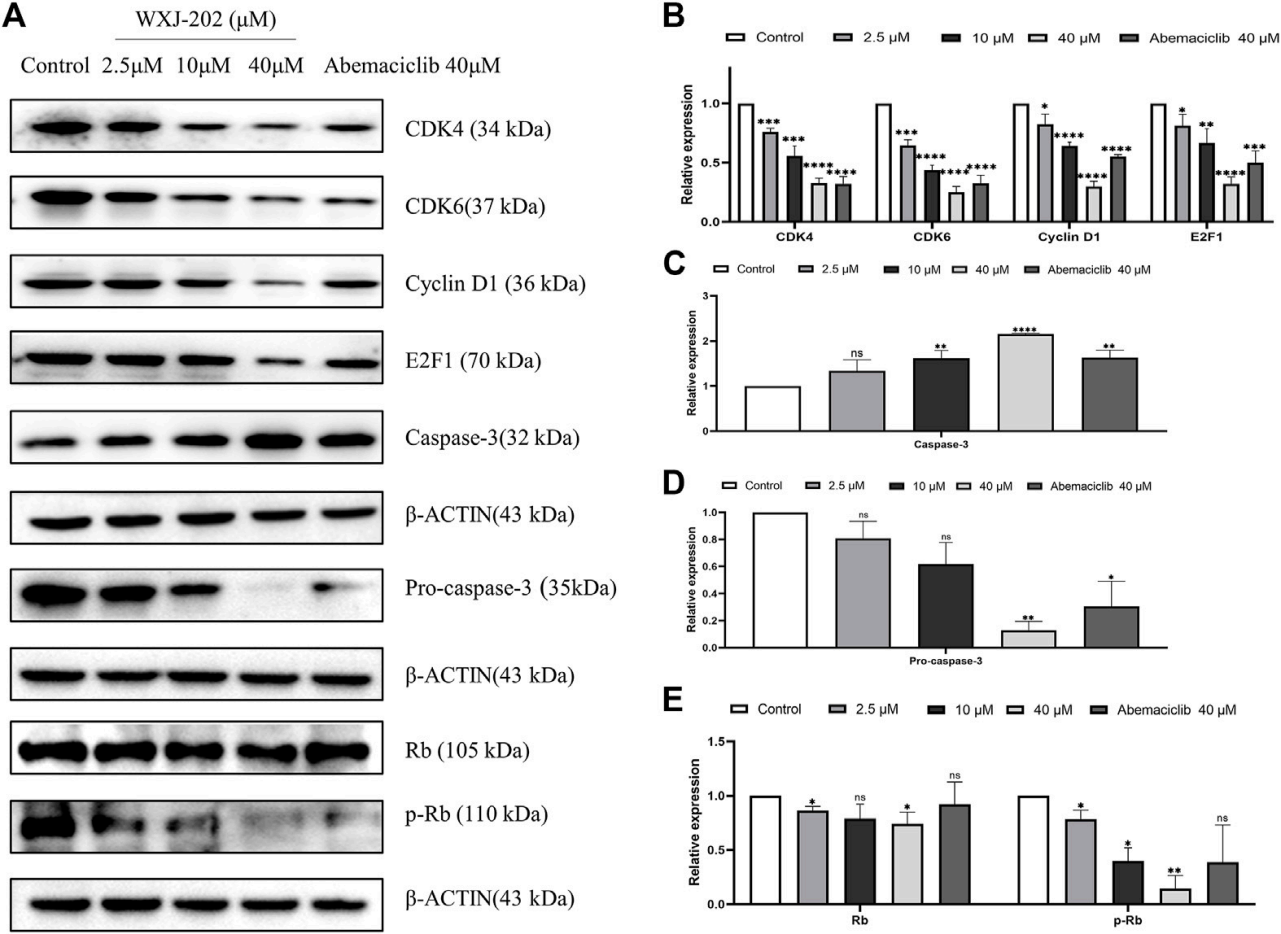
Effect of compound WXJ-202 on *CDK4/6*-*Rb*-*E2F* pathway-related proteins in MDA-MB-231 cells. **(A–E)** The expression of cell apoptosis-related proteins and cell cycle-related proteins in MDA-MB-231 cells was analyzed by Western blotting. Results are representative of at least three independent experiments showing similar results. *p < 0.05 vs. control, **p < 0.01 vs. control, ***p < 0.001 vs control, ****p < 0.0001 vs. control.

Our experimental results proved that the compound WXJ-202 could induce apoptosis and cycle arrest in MDA-MB-231 cells. To investigate more deeply the mechanism of the above-mentioned effects, we performed Western blot assays. The results showed that compound WXJ-202 down-regulated the expression of MDA-MB-231 cell cycle-related proteins *CDK4*, *CDK6*, *Cyclin D1*, and E2F1, and the *p-Rb*/*Rb* ratio was reduced in a dose-dependent manner (Figures 8A, B, E). *CDK4/6*-*Rb*-*E2F* is a classic signaling pathway that regulates the cell cycle. As confirmed by our experimental results, when *CDK4* and *CDK6* were inhibited, the expression of *p-RB* and *E2F1* was also reduced. Thus, the associated tumor cells were prevented from entering S-phase from G1-phase, thereby inhibiting the excessive proliferation of tumor cells and preventing the abnormal replication of tumor cells.

### 3.9 *In vivo* anti-proliferative activity of compound WXJ-202

**FIGURE 9:**
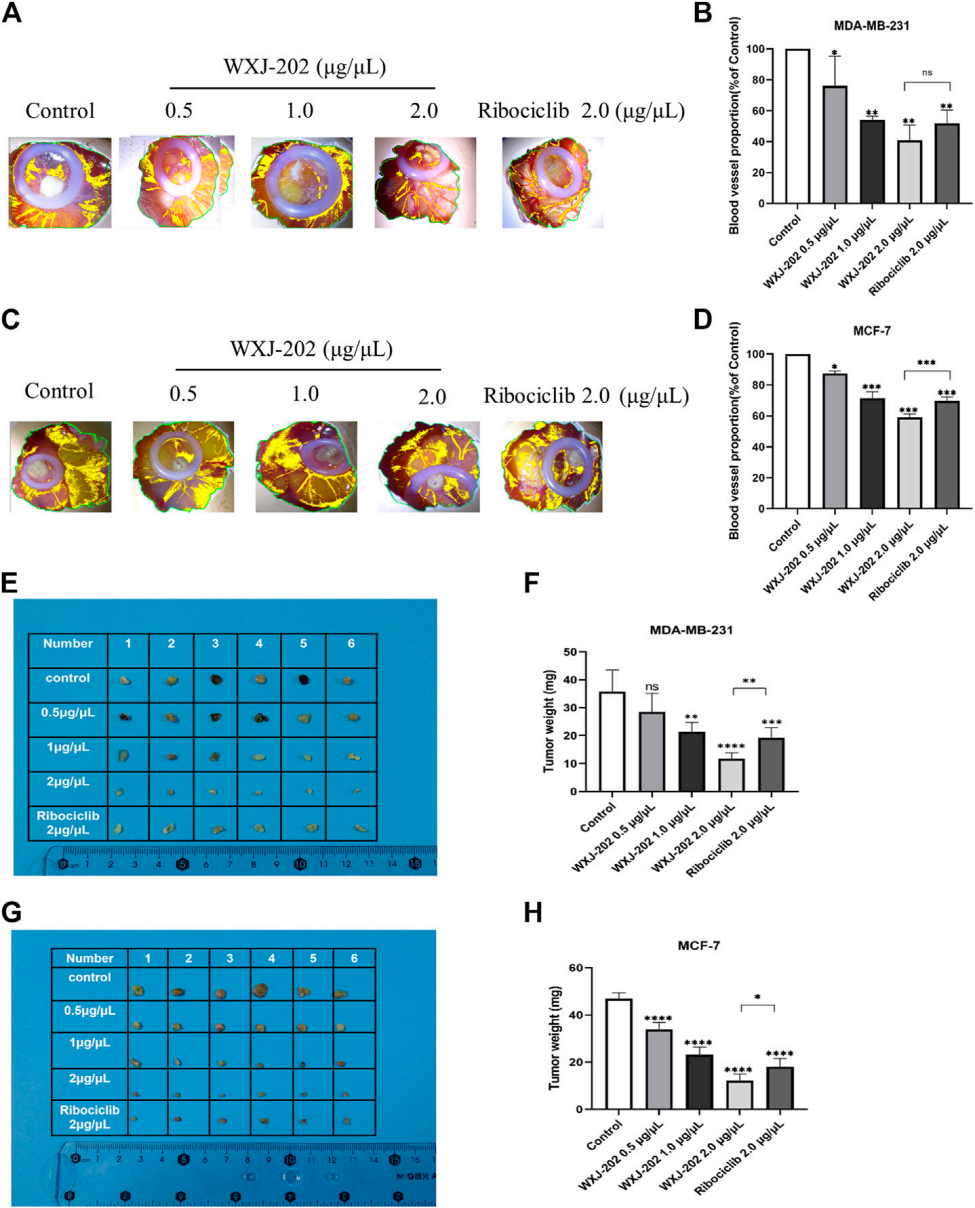
Effects of compound WXJ-202 and Ribociclib on the chicken embryo chorioallantoic membrane model. **(A–D)** Statistical graphs of the neo-vascular situation and the number of vessels (*n* = 3). **(E–H)** Statistical diagram of the growth of resected tumors and the weight of tumors in each group (*n* = 6). *p < 0.05 vs control, **p < 0.01 vs. control, ***p < 0.001 vs. control, ****p < 0.0001 vs. control.

To further investigate the *in vivo* antitumor activity of compound WXJ-202, we constructed chicken embryo transplant tumor models with MDA-MB-231 and MCF-7 cells. After treatment with the compound WXJ-202 at concentrations of 0.5 μg/μL, 1 μg/μL, and 2 μg/μL, the angiogenesis rates were (76.08 ± 19.13%, 87.45 ± 1.57%), (54.12 ± 2.39%, 71.58 ± 4.12%), and (40.78 ± 9.94%, 59.03 ± 2.18%), respectively, with significantly enhanced ability to inhibit tumor angiogenesis and stronger effect than the positive drug Ribociclib at the same concentration (51.82 ± 8.62%, 69.83 ± 2.40%) (Figures 9A–D). Meanwhile, our experimental results showed that weights of chicken embryo tumors were (28.50 ± 6.57 mg, 33.83 ± 3.06 mg), (21.30 ± 3.39 mg, 23.17 ± 3.19 mg), and (11.70 ± 2.16 mg, 12.17 ± 2.79 mg) after treatment with low, medium, and high concentrations of compound WXJ-202, compared with the control group (35.70 ± 7.81 mg, 47.00 ± 2.37 mg), respectively (Figures 9E–H). Our experimental results showed that the tumor growth inhibition ability was enhanced with increasing concentration of compound WXJ-202 administration. In conclusion, compound WXJ-202 significantly inhibited the growth of MDA-MB-231 and MCF-7 cells in chicken embryo transplanted tumors, reflecting its excellent anti-tumor cell proliferation activity *in vivo*.

## 4 Discussion

The cell cycle is an essential part of cellular life activity, and its proper conduct must be precisely and tightly regulated by regulatory factors at all levels (Carcagno et al., 2011). *CDKs* are essential regulators of the tumor cell cycle (Duster et al., 2022). *Cyclin D* activates *CDK4/6* to form the *CDK4/6*-*Cyclin D* complex, which phosphorylates a series of substrates, including *Rb* (Wagner and Gil, 2020). Phosphorylation of *Rb* releases the *E2F* transcription factor, which can activate and transcribe genes required for entry into the S phase (Pines, 1993; Sherr, 1995). Once the balance is disturbed by certain factors, cell proliferation can get out of control, thus promoting tumor progression. Breast cancer is the leading cause of death in women (Wu et al., 2022). There is a limited selection of clinically available chemotherapeutic agents for the treatment of TNBC, and their efficacy is often limited, such that continued exploration of novel drugs is critical for the treatment of TNBC (Liang et al., 2022). In this study, we designed and synthesized a novel Ribociclib derivative named WXJ-202 and further investigated its antitumor effects on MDA-MB-231 and MCF-7 cells. Palbociclib, Ribociclib, and Abemaciclib are *CDK4/6* inhibitors approved by the FDA for the treatment of breast cancer, but patients develop drug resistance with long-term use (Papadimitriou et al., 2022). WXJ-202 had significantly better toxic effects on MDA-MB-231 cells than the positive drugs Imatinib, Ribociclib, and Abemaciclib. Therefore, compound WXJ-202 had significant potential as a novel molecularly targeted agent for the treatment of TNBC.

*CDK4/6* overexpression results in abnormalities in the *CDK4/6*-*Rb*-*E2F* pathway, which is a core cause of the development of breast cancer (Lin et al., 2019). In our experiments, compound WXJ-202 was found to inhibit cell proliferation, migration, invasion, and colony formation, as well as induce apoptosis and G0/G1 phase cycle arrest of breast cancer cells. To investigate in depth the mechanism of apoptosis and cycle arrest effects induced by compound WXJ-202 in MDA-MB-231 cells, Western blot experiments were performed, and the results revealed that compound WXJ-202 led to the inhibition of *CDK4*, *CDK6*, *Cyclin D1*, and *E2F1* expression was inhibited. Meanwhile, the ratio of *p*-*Rb*/*Rb* also decreased with increasing concentration of WXJ-202. These results indicated that WXJ-202, after acting on breast cancer cells, could exert anti-tumor effects by inhibiting the expression of *CDK4* and *CDK6*, thus affecting the formation of *CDK4/6*-*Cyclin D1* complex and inhibiting the phosphorylation of *Rb*. In addition, the results also showed an increase in the apoptosis-related protein *Caspase*-*3* and a decrease in the expression level of *Pro*-*caspase*-*3*. This suggested that the compound WXJ-202 could exert anti-TNBC effects by interfering with the *CDK4/6*-*Rb*-*E2F* pathway. Uncontrolled *Cyclin D1*-*CDK4/6*-mediated *Rb* phosphorylation in tumor cells leads to sustained cell division and tumor growth (Filizoglu et al., 2022). Compound WXJ-202 significantly blocked the phosphorylation of *Rb* and the blocking effect was superior to that of the positive drug Abemaciclib at the same concentration. Consistent with the above findings, compound WXJ-202 significantly inhibited tumor growth in the chicken embryo chorioallantoic model. These results emphasized that compound WXJ-202 was a potential targeted agent for the treatment of TNBC with excellent and significant anti-tumor cell proliferation activity.

In conclusion, our research demonstrated that WXJ-202 induced apoptosis and cycle arrest through affected *CDK4/6*-*Rb*-*E2F* pathway-associated proteins, producing antitumor effects on breast cancer cells *in vitro and in vivo*. WXJ-202, as a novel Ribociclib derivative, provided the basis for exploring drugs for the treatment of TNBC.

## Data Availability

The raw data supporting the conclusions of this article will be made available by the authors, without undue reservation.
