# Supplementary figures and images for: WXJ-202, a novel Ribociclib derivative, exerts antitumor effects against breast cancer through CDK4/6
Source: Front Pharmacol. 2023 Jan 19;13:1072194. doi: 10.3389/fphar.2022.1072194 (PMC9894725; doi:10.3389/fphar.2022.1072194)

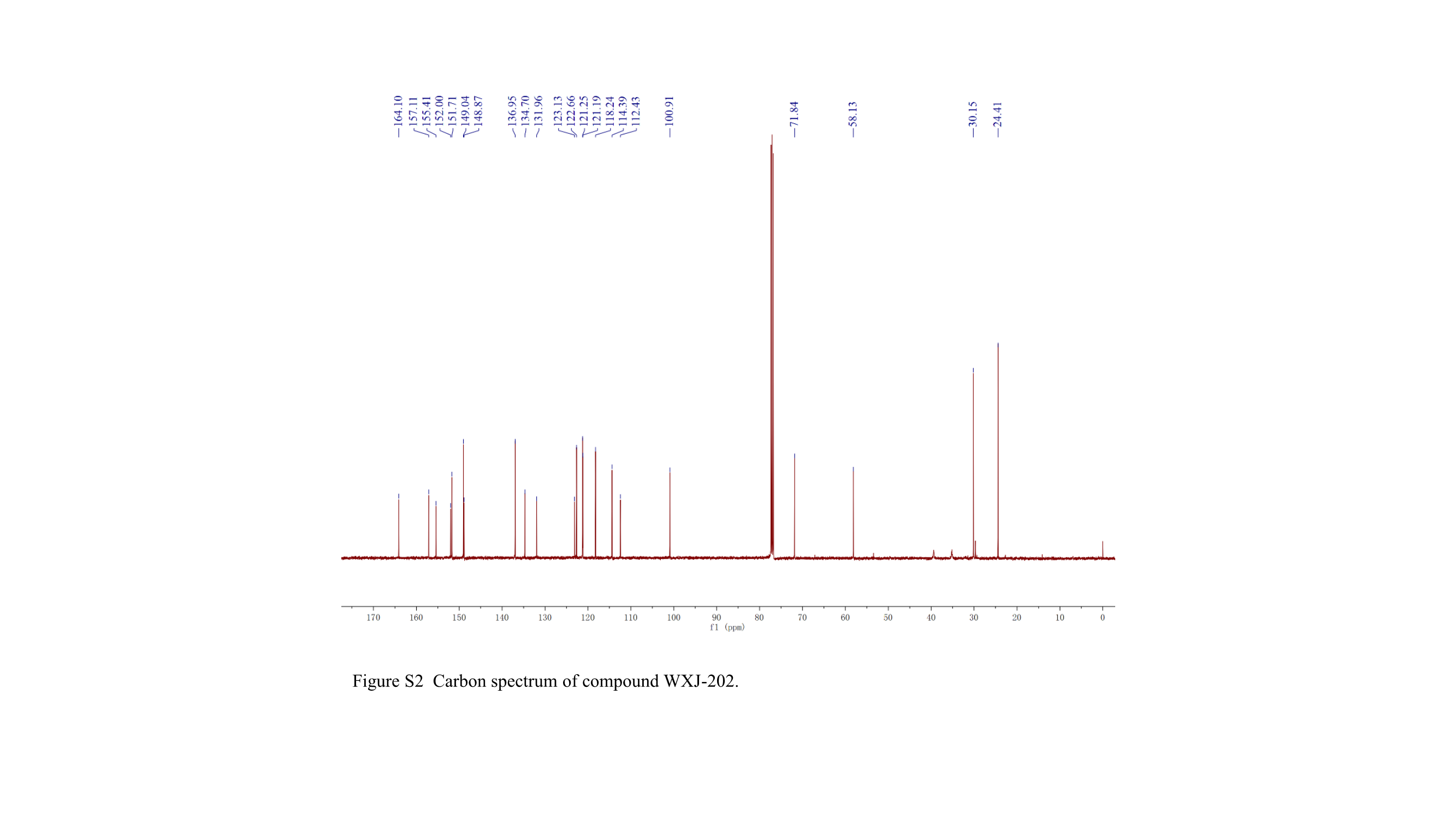

Supplement: Supplementary file 1 [file Image2.TIF]

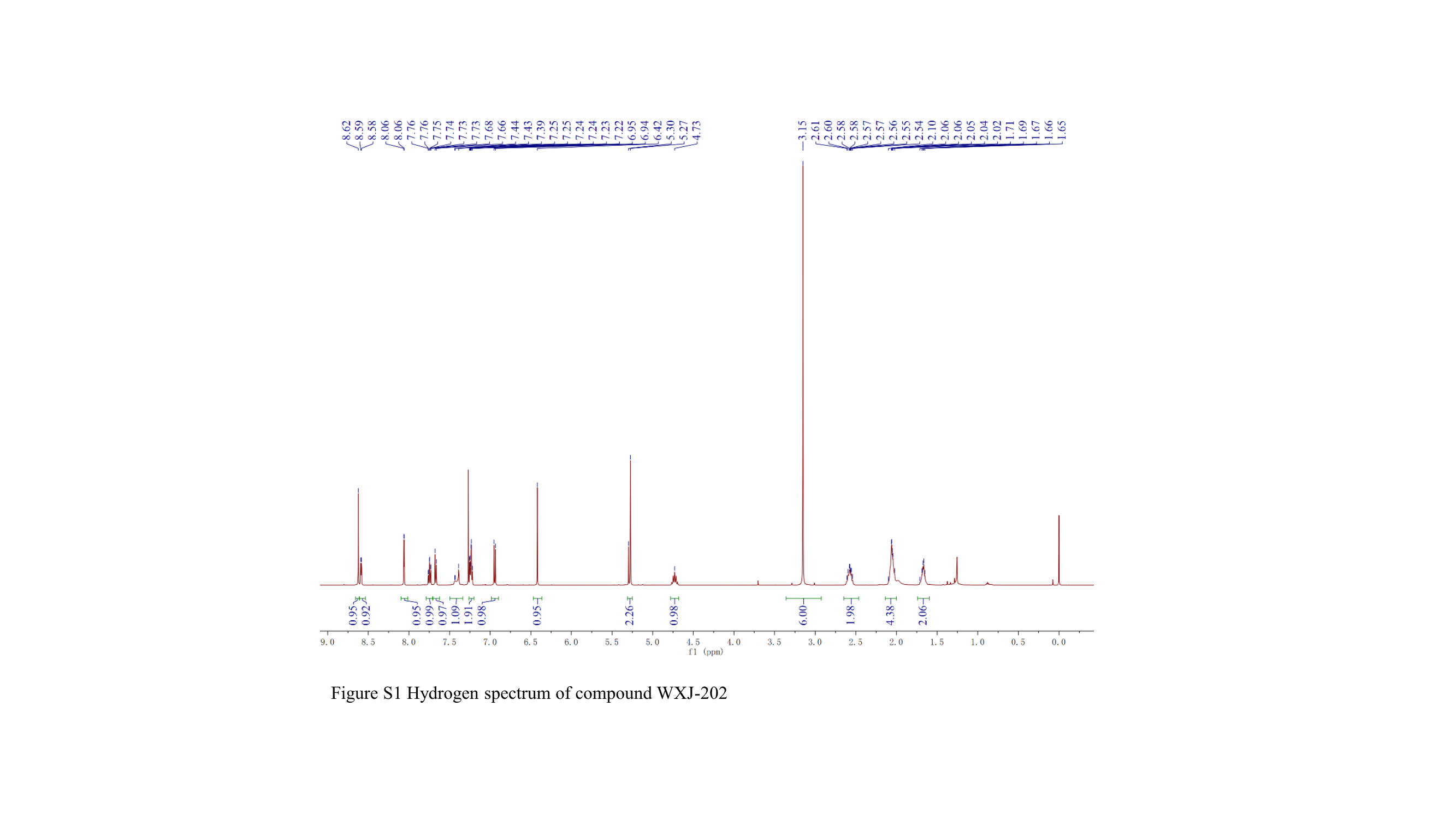

Supplement: Supplementary file 2 [file Image1.TIF]
